# 

**Published:** 1842-04-01

**Authors:** 


### BIBLIOGRAPHICAL RECORD.

1. The Dublin Journal of Medical Science. No. 60. Jan. 1842.

---

2. Third Annual Report of the Registrar-General of Births, Deaths and Marriages in England, with Appendices. Presented to both Houses of Parliament. Clowes, London, 1842.

---

3. The Retrospect of Practical Medicine and Surgery. Being a half-yearly Journal, &c. Edited by W. BRAITHEWAITE, Esq. &c. &c. Vol. II. July—December, 1841. Simpkin and Marshall, London, Christmas, 1841.

---

4. Natural History of Man. By JAMES COWLEY PRICHARD, M.D. F.R.S. Illustrated with many coloured plates, engraved on steel, and interspersed with numerous wood-cuts. Nos. 1, 2, and 3, price 2s. 6d. each. To be completed in ten monthly parts, forming a thick volume. Bailliere, Regent-street, Jan. 1842.

☞ *This may be considered a national, or rather a cosmopolitan work.*

---

5. Atlas illustrative of the Anatomy of the Human Body. By J. CRUVEILHIER, M.D. The figures drawn by Em. Beau, with explanations by C. Bonany, M.D. Parts 1 to 5, inclusive, price 2s. 6d. plain —coloured 5s. Bailliere, Regent-street, Jan. 1842.

☞ *An admirable work.*

---

6. On Rheumatism in its various forms, and on the Affections of Internal Organs, more especially the Brain and Heart to which it gives rise. By RODERICK MACLEOD, M.D. Physician to St. George's Hospital. 8vo. pp. 164. Longman and Co. 1842.

---

7. Interesting Facts connected with the Animal Kingdom; with some Remarks on the Unity of our Species. By JOHN CHAS. HALL, M.D. &c. 8vo. pp. 301. Whittaker and Co. London, 1842.

---

8. Elements of General and Minute Anatomy of Man and the Mammalia, chiefly after original researches. By F. R. GERBER, Prosector in the University of Berne. To which are added, Notes and an Appendix, &c. By GEORGE GULLIVER, F.R.S. In two volumes, Text, Appendix, and Atlas. Bailliere, Regent-street, 1842.

---

9. Facts and circumstances relating to a Case of Compound Fracture, prosecuted, &c. at Cortland Village, Cortland County, United States. By A. B. SKIPMAN, M.D.

☞ *Another melancholy exhibition of the rancour, hatred, jealousy, and illiberality pervading our unhappy Profession!!*

---

10. Elements of Materia Medica and Pharmacy. By O'BRYEN BELLINGHAM, M.D. Lecturer on Materia Medica, &c. &c. Edited by ARTHUR MITCHELL, M.D. &c. Part 1, pp. 302. Dublin, Fannin and Co. London, Longman and Co. Jan. 1842. Price 6s.

---

11. Observations on the Religious Delusions of Insane Persons, and on the practicability, safety, and expediency of imparting to them Christian Instruction, &c. &c. By NATHANIEL BINGHAM, M.R.C.S. 8vo. pp. 213. J. Hatchard and Co. Piccadilly, 1841.

---

12. An Essay on the Influence of Constitution in the production of Disease. By ED. OCT. HOCKEN, M.D. &c. Duodecimo, pp. 64. Highley, London, 1842. Price 2s. 6d.

---

13. A Practical Treatise on the Diseases of Children. By JAMES STEWART, M.D. 8vo. pp. 547. New York, 1841.

☞ *The most complete and erudite treatise on the subject in the English language.*

---

14. The Medical Times, for the quarter ending 31st Dec. 1842.—*In exchange.*

---

15. The London and Edinburgh Monthly Journal of Medical Science. Conducted by J. R. CORMACK, M.D. Ed. Feb. 1842.

---

16. A Treatise on Dislocations and Fractures of the Joints. By Sir A. Cooper, Bt. A new Edition, much enlarged. Edited by Mr. Bransby B. Cooper, F.R.S. Surgeon to Guy's Hospital. Octavo, pp. 576, with numerous plates, price 20s. Churchill, 1842.

☞ *With numerous additions, this edition is not half the price of the original quarto edition.*

---

17. An Inquiry into the Nature and athology of Granular Disease of the Kidney, and its mode of action in producing Albuminous Urine. By George Robinson. 8vo. pp. 79. Churchill, London, Feb. 1842.

---

18. Catalogue of the Preparations illustrative of Normal, Abnormal, and Morbid Structure, human and comparative, constituting the Anatomical Museum of George Langstaff, M.R.C.S. &c. Octavo, pp. 518. Churchill, 1842.

---

19. On the Treatment of Stone in the Bladder, by Medical and Mechanical Means. By R. Willis, M.D. &c. 8vo. pp. 183. Bailliere, 1842.

---

20. Contributions to Aural Surgery. No. 4. On Deafness from morbid conditions of the Mucous Membrane of the Stomach, Throat, and Ear, the effect of Cold, Dyspepsia, Scarlatina, Measles, &c. By James Yearsley, M.R.C.S. 8vo. pp. 56. Nesbit and Co. 1842.

---

21. Spinal and Nervous Diseases, Rheumatism and Paralysis:—or Cases and Observations illustrating an improved Treatment. By John Hey Robertson, M.D. 8vo. pp. 119. Glasgow, 1842.

---

22. A Practical Treatise on Venereal Diseases; or critical and experimental researches on Inoculation, applied to the Study of these Affections, &c. &c. By Ph. Ricord, M.D. Translated from the French by Henry Pilkington Drummond, M.D. 8vo. pp. 384. Longman and Co. 1842.

---

23. A Series of Anatomical Plates, &c. By Jones Quain, M.D. Fascic. 95, 96, and 97. Division, Bones and Ligaments. Taylor, London, 1842.

---

24. Anatomie Pathologique du Corps Humaine. Cruveilhier. Bailliere, Regent-street, 1842.

25. A Suggestion for the Cure of Cholera. By Lieut. H. Congreve, Madras Artillery. 8vo. pp. 48. Richardson London, 1841.

---

26. The Administration of Medical Relief to the Poor, under the Poor-law Amendment Act, and other Legislative Provisions for the Public Health, &c. &c. suggested for insertion in the contemplated Bill for the further Amendment of the Poor-Laws. 8vo. pp. 136. Sherwood and Co. 1842.

---

27. Hydropathy; or the Cold Water Cure, as practised by Vincent Priessnitz, at Gräeffenberg, in Silesia. By R. T. Claridge, Esq. 8vo. pp. 316. James Madden, 1842.

---

28. Physiology for the Public, &c. in a series of Lectures. By G. T. Hayden, A.B. M.B. of Trinity College Dublin. No. 4, Price 1s.

☞ *The author sustains the popular interest of the subject, both in matter and manner.*

---

29. Principles of Surgery. By James Syme, F.R.S.E. Professor of Clinical Surgery, Ed. Third edition enlarged, and illustrated with 64 wood-cuts and 14 plates. 8vo. pp. 508. Bailliere, London, 1842.

---

30. A Memoir of William Maclure, Esq. late President of the Academy of Natural Sciences of Philadelphia. By Dr. Morton, of Philadelphia. 1841.

☞ *An eloquent eloge on departed merit.*

---

31. A Pentaglot Dictionary of the terms employed in Medicine, &c. Part 1. with the leading terms in French, followed by the synonimes in the Greek, Latin, German, and English: explanations in English, and copious illustrations in the different languages. Part II. a German, English, French Dictionary, comprehending the scientific German terms of the preceding Part. By Shirley Palmer, M.D. One large vol. 8vo. 656 pages in double cols. Longman and Co. 1841.

---

32. Pharmaceutical Journal and Transactions. Edited by Jacob Bell. No. 9, March 1, 1842.

---

33. Remarks on Hypochondriasis. By H. P. Roberts, Esq. Read before the London Medical Society.

34. Statistical Reports of the Health of the Navy for the years 1830—36 inclusive. Part 2, Cape of Good Hope—West Coast of Africa—East Indies. Ordered to be printed. Oct. 1841. Price 3s. 9d.

---

35. The Transactions of the Provincial Medical and Surgical Association. Instituted 1832. Vol. X. Churchill, London, 1842.

☞ *In our next.*

---

36. The Anatomy of the Urinary Bladder and Perineum of the Male. Illustrated by Engravings, with Physiological, Pathological, and Surgical Observations. By Alex. Monro, M.D. Professor of Anatomy, &c. 8vo. pp. 90, with numerous plates. Edinburgh, Maclachlan, Stewart and Co. 1842.

---

37. A Dispensatory, or Commentary on the Pharmacopœias of Great Britain; comprising the natural history, description, chemistry, pharmacy, actions, uses, and doses of the Articles of the Materia Medica. By Robert Christison, M.D. F.R.S.E. &c. &c. 8vo. pp. 978, with index, &c. Edinburgh, Adam and Charles Black, 1842.

---

38. Lectures on the Diseases of the Urinary Organs. By Sir B. Brodie, Bart. Third edition, with alterations and additions. Longman and Co. 1842.

---

39. An Investigation of the present unsatisfactory and defective State of Vaccination, &c. &c. addressed to Dr. George Gregory. By Thomas Brown, Esq. 8vo. pp. 139. Maclachlan, Stewart, and Co. Edinburgh, 1842.

---

40. The Hunterian Oration, delivered [illegible] the Royal College of Surgeons, Feb. 14 1842. By G. G. Babington, Esq. Surgeo[illegible] to St. George's Hospital.

☞ *One of the most eloquent orati[illegible] that have been read at the College for m[illegible] years.*

---

41. Observations on the Mineral Springs of Harrogate. By William Bennett, M.D. 8vo. stitched, pp. 11.

---

42. Principles of Human Physiology with their chief applications to Pathology, Hygiene, and Forensic Medicine. Especially designed for the use of Students. By W. B. Carpenter, M.D. Lecturer on Physiology, in the Bristol Medical School. 8vo pp. 680. Churchill, London, March 1842.

---

43. A Practical Treatise on Nervous Impediments of Speech, Stammering, and Debility of the Vocal Organs, &c. with Remarks on the irrational Surgical Operations, &c. &c. By Joseph Poett, Esq. &c. 5th Edition, duodecimo, pp. 100. Highley, 1842.

---

44. Summary of the Weekly Tables of Mortality (Metropolis) for 1841. Population 1,870,727. By authority of the Registrar General.

